# A PDMS-based microfluidic platform enabling dual biochemical and electric-field stimulation for modeling sensory neuron-intervertebral disc crosstalk

**DOI:** 10.64898/2026.07.29.741573

**Published:** 2026-07-30

**Authors:** Olanrewaju Akande, Sade W. Clayton, Liufang Jing, David Duong, Bryce Stottlemire, Ryan Potter, Mohammadjafar Hashemi, Addison Liefer, Nathaniel Huebsch, Lori Setton, Simon Y. Tang, Cory Berkland

## Abstract

Inflammation-driven increases in nociception are prominent in pain pathologies associated with intervertebral disc (IVD) degeneration yet are difficult to model in vitro. Since neurons are exposed to multi-modal stimuli in vivo, it is critical for these exposures to be conserved in an in vitro test system. We developed a polydimethylsiloxane (PDMS)-based microfluidic platform to interrogate peripheral sensory neurons (SNs) in the presence of conditioned media from nucleus pulposus cells from the degenerated IVD, to model a potential impact of IVD cells’ secretome on pain sensing. Our platform enables controlled perfusion of cell-derived biochemical cues alongside a defined homogeneous electric field (EF) and supports real-time optical analysis. Computational modeling, fluid perfusion experiments, and conductivity measurements confirmed stable fluid transport and tunable homogeneous EF generation within the device. As proof of concept for neuronal stimulation, neuroblastoma (N2a) cells loaded with a fluorescent Ca ^2+^ indicator exhibited a 56% increase in Ca ^2+^ transient activity when exposed to media from degenerated IVDs, concomitant with increased IVD-derived IL-1β production. Importantly, EF-stimulated Ca ^2+^ transients increased in SNs derived from human induced pluripotent stem cells when exposed to conditioned media from primary human IVD cells, demonstrating the translation of this model system to human cells. Together, these results establish a versatile platform that enables controlled and simultaneous exposure to biochemical and electrical stimuli to quantify inflammation-driven peripheral neuronal hyperexcitability in tissue–neuron crosstalk.

## Full Text Availability

The license terms selected by the author(s) for this preprint version do not permit archiving in PMC. The full text is available from the preprint server.

